# ‘It’s tough. It is hard’: A qualitative interview study of staff and volunteers caring for hospice in-patients with delirium

**DOI:** 10.1177/02692163231170655

**Published:** 2023-05-02

**Authors:** Imogen Featherstone, Najma Siddiqi, Lesley Jones, Eleonora Coppo, Trevor Sheldon, Annmarie Hosie, Anna Wolkowski, Shirley H Bush, Johanna Taylor, Andrew Teodorczuk, Miriam J Johnson

**Affiliations:** 1 Department of Health Sciences, University of York, York, UK; 2 Hull York Medical School, University of Hull, Hull, UK; 3 Cardinal Massaia Hospital of Asti, Asti, Piemonte, Italy; 4 Wolfson Institute of Population Health, Barts and The London School of Medicine and Dentistry, Queen Mary University, London, UK; 5 School of Nursing, The University of Notre Dame Australia, Sydney, NSW, Australia; 6 The Cunningham Centre for Palliative Care, St Vincent’s Health Network, Sydney, NSW, Australia; 7 IMPAACT, Faculty of Health, University of Technology Sydney, Sydney, NSW, Australia; 8 Dove House Hospice, Hull, UK; 9 Department of Medicine, Division of Palliative Care, University of Ottawa, Ottawa, ON, Canada; 10 Bruyere Research Institute, Ottawa, ON, Canada; 11 Ottawa Hospital Research Institute, Ottawa, ON, Canada; 12 Faculty of Medicine, The University of Queensland, Brisbane, QLD, Australia; 13 The Prince Charles Hospital, Metro North Mental Health, Brisbane, QLD, Australia; 14 School of Medicine and Dentistry, Griffith University, Southport, QLD, Australia; 15 Wolfson Palliative Care Research Centre, Hull York Medical School, University of Hull, Hull, UK

**Keywords:** Delirium, palliative care, hospices, qualitative research, psychological distress

## Abstract

**Background:**

Delirium is a distressing condition often experienced by hospice in-patients. Increased understanding of current multidisciplinary care of delirium is needed to develop interventions in this setting.

**Aim(s):**

To explore hospice staff and volunteers’ practice, its influences and what may need to change to improve hospice delirium care.

**Design:**

Qualitative interview study using behaviour change theory from a critical realist stance.

**Setting/participants:**

Thirty-seven staff, including different professional groups and roles, and volunteers were purposively sampled from two in-patient hospices.

**Results:**

We found that participants’ practice focus was on managing hyperactive symptoms of delirium, through medication use and non-pharmacological strategies. Delirium prevention, early recognition and hypoactive delirium received less attention. Our theoretically-informed analysis identified this focus was influenced by staff and volunteers’ emotional responses to the distress associated with hyperactive symptoms of delirium as well as understanding of delirium prevention, recognition and care, which varied between staff groups. Non-pharmacological delirium management was supported by adequate staffing levels, supportive team working and a culture of person-centred and family-centred care, although behaviours that disrupted the calm hospice environment challenged this.

**Conclusions:**

Our findings can inform hospice-tailored behaviour change interventions that develop a shared team understanding and engage staff’s emotional responses to improve delirium care. Reflective learning opportunities are needed that increase understanding of the potential to reduce patient distress through prevention and early recognition of delirium, as well as person-centred management. Organisational support for adequate, flexible staffing levels and supportive team working is required to support person-centred delirium care.

**What is already known about the topic?**

- Delirium is a distressing condition often experienced by hospice in-patients.
- Tailored evidence-based interventions for delirium in hospices need to be developed.

**What this paper adds**

- Hospice staff and volunteers’ practice predominantly focused on managing hyperactive symptoms of delirium, rather than delirium prevention or early recognition.
- Our theoretically informed analysis identified this focus was influenced by staff and volunteers’ emotional responses to the distress associated with hyperactive symptoms of delirium underpinned by the lack of a shared team understanding of delirium care.
- Non-pharmacological delirium management was supported by adequate staffing levels, supportive team working and a culture of person-centred care, although behaviours that disrupted the calm hospice environment challenged this.

**Implications for practice, theory or policy**

- Organisational support for adequate, flexible staffing levels and supportive team working is required to support person-centred delirium care.
- Reflective learning opportunities could increase understanding of the potential to reduce patient distress through prevention and early recognition of delirium, as well as person-centred management.
- These findings can inform behaviour change interventions, tailored to hospices, that develop a shared team understanding and engage staff’s emotional responses to improve delirium care.

## Introduction

Delirium is a distressing condition often experienced by hospice in-patients.^1,2^ It is characterised by acute, fluctuating disturbances in attention, awareness and cognition, stemming from underlying physiological causes.^3^ Palliative care patients most commonly experience the under-recognised hypoactive subtype, and report as much distress as those with hyperactive symptoms.^1,4–7^

There is little research into how to prevent and manage delirium in hospice in-patients.^8–12^ In the UK context, in contrast to other countries,^13,14^ hospices are independent, charitably funded organisations which provide in-patient symptom management and end-of-life care, amongst other palliative care services. In hospital settings multicomponent interventions can reduce delirium incidence by a third.^15^ Clinical guidelines (not palliative care-specific) recommend delirium screening, interventions targetting delirium risk factors, assessment and treatment of underlying causes, non-pharmacological strategies to support patients and family involvement in care.^16,17^ Systematic reviews demonstrate little high quality evidence supporting routine use of medication for delirium.^18,19^ Evidence and guidelines from other settings are useful, but differences in hospice patients, organisation and culture need to be taken into account.

Qualitative studies provide useful insights into how delirium care practice in palliative care settings aligns with, or differs from, evidence and guidelines, and the influences on this. Our qualitative review found most research explored nurses’ perspectives and focused on delirium management rather than prevention or early identification.^2^ Interview studies with nurses identified they lacked knowledge and skills in delirium recognition, assessment and management.^20–22^ Use of medication was triggered by patients’, families’ and clinicians’ distress, and time and staffing pressures.^20,23–25^ Person-centred approaches and family involvement were important enablers for good delirium care.^22–24,26–31^

This study addresses UK in-patient hospice care. A more comprehensive understanding is needed of the delirium practice behaviours of multidisciplinary hospice teams operating within organisational cultures distinct from the NHS and other palliative care contexts. Structured analysis of the influences on these behaviours is necessary to inform the development of tailored interventions to improve delirium care in this setting. Our study objectives were to explore:

1. Hospice staff and volunteers’ practices in delirium prevention, recognition, assessment and management.
2. The individual and organisational influences upon that practice.
3. Practice strengths, and what may need to change, to inform the development of tailored interventions to improve delirium care in hospices.

## Methods

We report this study according to the Standards for Reporting Qualitative Research (SRQR).^32^

### Design

This qualitative interview study is part of a mixed-methods research programme to design an intervention to improve hospice delirium practice.^33,34^ We used thematic analysis, informed by behaviour change theory; the COM-B (Capability, Opportunity, Motivation- Behaviour) framework and Behaviour Change Wheel.^35,36^ COM-B theorises that Capability (physical and psychological), Opportunity (physical and social) and Motivation (automatic and reflective) interact to generate behaviour (See Table 1). For the purpose of the study this enabled structured analysis of the influences on staff and volunteers’ delirium care behaviours to target for change, and techniques useful for interventions. We used a critical realist approach, based on the assumption of a shared reality, our understanding of which is mediated by our cultural contexts.^37^

**Table 1.** Definitions of the components of the COM-B model.^35,38^.

COM-B component	Definition
Physical capability	Physical skill, strength or stamina
Psychological capability	Knowledge, understanding or psychological skills, strength or stamina to engage in the necessary mental processes
Physical opportunity	Opportunity afforded by the environment involving time, resources, locations, cues, physical ‘affordance’
Social opportunity	Opportunity afforded by interpersonal influences, social cues and cultural norms that influence the way we think about things.
Reflective motivation	Reflective processes involving plans (self-conscious intentions) and evaluations (beliefs about what is good and bad)
Automatic motivation	Automatic processes involving emotional reactions, desires (wants and needs), impulses, inhibitions, drive states and reflex responses.

### Setting

Two independent third-sector UK hospices with in-patient units (Hospice 1 = 21 beds; Hospice 2 = 18 beds) were included. Neither had a delirium guideline, delirium screening tool or patient/family information in use at the time of data collection.

### Participants

Eligible participants were consenting hospice staff members (doctors, nurses, allied health professionals, health care assistants, domestic workers, managers, fund-raising staff, board members) and volunteers. There were no specific exclusion criteria.

### Sampling and recruitment

Participants were purposively sampled to include the perspectives of these different groups. Participants with non-patient facing roles were included to gain understanding of organisational level factors, hospice culture, staff support systems and resources.

Potential participants were informed about the study through emails, posters and researchers (IF, LJ) attending staff meetings. Those interested contacted the research team directly.

‘Information power’ was evaluated during the study to inform sample size, guided by the criteria: breadth of study aim; sample specificity; use of theory; quality of interview dialogue; analysis strategy.^39^ This approach is congruent with our use of theoretically-informed thematic analysis.

### Data collection

Semi-structured individual interviews were conducted in-person and by telephone (IF, LJ, October 2017-April 2018) using an interview guide and case vignettes of patients with hypoactive and hyperactive delirium developed by the research team (IF, LJ, NS, MJ, AH) with public involvement group input (Supplemental File 1). Pilot interviews were conducted at another hospice site. Interviews were audio-recorded and transcribed verbatim.

Quantitative demographic information was collected on participants’ age, sex, role and years of palliative care experience.

### Data analysis

Thematic analysis was used to generate, analyse and interpret themes from the interview data,^36^ supported by Nvivo software.^40^ We used elements of both ‘theory-driven’ and ‘data-driven’ approaches.^36,41^ Coding was informed by delirium guidelines but a pre-defined coding framework was not used. The development of descriptive themes was based upon participants’ experiences of delirium care, and the COM-B framework then used to support development of analytical themes.

Two researchers (IF, EC) initially carried out independent line by line coding of the same interviews, before developing a shared coding scheme. They then coded separate interviews, regularly reviewing the developing analysis together with a third reviewer, LJ. Descriptive themes and subthemes were developed by sorting and collating codes, reviewing their content and exploring relationships between them using memos and mindmaps.

COM-B was then used to support structured analysis of the influences on staff and volunteers’ delirium care behaviours, strengths and what may need to change (written by IF, reviewed by NS, MJ, TS, LJ and the public involvement group).^35^ Trustworthiness and credibility was enhanced by the use of ‘constant comparison’ and the involvement of several researchers.^42^

Reflexivity: Research team members have research and clinical experience in delirium, palliative care and qualitative methods. Our understanding of current guidelines, and public involvement input informed the design and conduct of this study, and our interpretation of the findings.

Ethical considerations: This study was approved by Hull York Medical School Ethics Committee (24.07.17, Ref: 1717) and hospice institutional permissions gained. Written informed consent was gained for face-to-face interviews, recorded verbal consent for telephone interviews.

## Findings

Participants: Thirty-seven participants were interviewed. Mean interview duration was 35 min (Range 15–63 min). See Table 2 for participant characteristics.

**Table 2.** Participant characteristics.

Total participants	37
Hospice 1	20
Hospice 2	17
Age (years)	Range = 21–73
	Median = 54.5
	Interquartile range (IQR) = 14.5 years
Sex (male/female)	*M* = 3, *F* = 34
Palliative care experience (years)	Range = 1–26
	Median = 9
	IQR = 10.5
*Participant role*	
Health care assistants	9
Nurses	8
Doctors	6
Volunteers	5
Domestic workers	2
Fundraisers	2
Board members	2
Occupational therapists	1
Physiotherapists	1
Senior managers	1

Thematic analysis: An over-arching theme was, ‘A reactive approach to hyperactive symptoms of delirium’. This expresses how participants’ practice focused on managing hyperactive symptoms. Other aspects of care, particularly prevention, early recognition and hypoactive delirium, received much less attention. We used COM-B to explore the influences driving this reactive approach to delirium care, as well as the influences on *how* hyperactive symptoms were managed. (See Table 3). Due to the breadth of scope of the COM-B concepts, all of our findings fitted with this theoretical framework.

**Table 3.** Themes, subthemes and main COM-B concepts.

Overarching theme: A reactive approach to hyperactive symptoms of delirium	
Major themes and subthemes	COM-B concepts
1. ‘You’re really fighting for them’: Emotional responses to delirium are powerful drivers of care a. Compassion and empathy in response to patients’ and families’ distress b. Stress in managing the patient’s delirium and its impact on others	Automatic motivation/social opportunity
2. Varied understanding of delirium influences the reactive focus on managing hyperactive symptoms a. Under-recognition of delirium as a medical condition b. ‘I don’t think you can. . .prevent it really’: Lack of shared understanding of delirium prevention c. Varied understanding of modifiable causes of delirium d. Understanding of the role of medication for delirium symptom management e. The need for a shared team understanding of delirium care	Psychological capability/reflective motivation
3. Hospice culture and environment influence person-centred delirium care a. ‘Patients and their families are at the centre’: Person-centred and family-centred approach to delirium care b. ‘If we do need extra staff we usually do get extra staff’: Time and staffing levels enable person-centred delirium care c. Supportive multidisciplinary team working	Social/physical opportunity

**1.** **‘You’re really fighting for them’: Emotional responses to delirium are powerful drivers of care (Automatic motivation/Social Opportunity)**

Staff and volunteers’ emotional responses to patients experiencing delirium and their families, were a strong influence on their focus on managing hyperactive symptoms.

a. Compassion and empathy in response to patients’ and families’ distress

Participants involved in direct patient care, including doctors, nurses, HCAs, therapists and volunteers, described patients with hyperactive symptoms of delirium experiencing visible distress, anxiety and fear,

People having hallucinations or people not knowing where they are or what’s happening to them so they get really stressed and panicky. (P31, HCA)

Participants’ empathy helped them to be compassionate towards patients who were aggressive,

Empathy and compassion. . .people that work here sympathise with the person. . .because they’ve not created that situation knowingly. (P23, Volunteer)

It must be so awful. . .It’s like if. . . I was saying something to you and you were saying, sorry, I can’t understand you, what do you mean; and yet I think I’m saying something perfectly normal. . .there’s no wonder they get so. . . angry. (P06, Nurse)

Many participants described how distressing the changes in patient’s behaviour can be for family members,

Because things aren’t as normal and they’re not as used to. . . dad turning round and f’ing and blinding and, and taking all his clothes off. . .and it’s very, very distressing for. . . relatives. (P06, Nurse)

Their empathy for the patient and family’s distress could be a powerful motivation to act,

Obviously that person, that family, are your absolute focus. . . I think to relate to a situation and put yourself in that poor wife’s situation is the way that you, you’re really fighting for them. It sounds quite dramatic (laughs) but it is at the time. (P22, Nurse)

b. Stress in managing the patient’s delirium and its impact on others

Participants, particularly those who spent most time with patients, described experiencing intense stress due to managing the distress of patients with hyperactive symptoms and their families, and the impact on other hospice patients and their families. Nurses and HCAs described working with patients with hyperactive symptoms as emotionally and physically draining:

It was almost the whole shift, and you. . . could physically feel the tension in your own body. . . you’re exhausted at the end of it, you’re exhausted. (P22, Nurse)

They reported experiencing anxiety, distress and sometimes fear,

We have had some. . .that they get really threatening where we are frightened. (p7, HCA)

Doctors and nurses described frustration when they were unable to resolve delirium or comfort patients close to the end of life, which was central to their role and values,

Sometimes you feel a bit helpless because you’re thinking you’re doing as much as you can. . .you’re really trying but they’re not getting settled; and. . . it is a bit heart-breaking sometimes because. . .our whole aim is to try and give a peaceful and comfortable death as much as you can, so that’s our ultimate aim, but sometimes you feel frustrated as well because. . . you feel like you’re just not getting there, no matter what you’re trying to do, and. . . it can be upsetting. (P08, Nurse)

Staff and volunteers involved in direct patient care were not only trying to reduce the distress of agitated patients and their families, but also the impact on other patients and their families,

It’s trying to contain. . . the agitation without it upsetting other patients, and. . .that’s what can be quite hard sometimes. (P33, HCA)

The stress that staff experienced was cumulative, as they tried to meet everybody’s needs,

It’s tough, it is hard; and to do the best that you can for everybody, you know, like piggy in the middle. (P22, Nurse).

The desire to maintain a calm, welcoming environment, a highly valued cultural norm in the hospices, exerted further pressure on staff to control the disruptive behaviours of patients with hyperactive symptoms which challenged this.

So much work is done to make it a welcoming, a homely atmosphere. (Fundraiser, P17)

That’s what struck me about when I came here. . .It’s a place of calmness and peace. . .gives you that feeling of utter calmness, not only to the person, but to families as well. (P28, Volunteer)

Obviously this does become a problem when we have somebody shouting out and we have poorly patients in, in close proximity to them, and it’s not unknown for people to complain and their relatives to complain. (P6, Nurse)

This board member expressed that seeing an agitated patient could negatively affect visitors’ views of the hospice,

It would not help them to feel comfortable, might put them off from wanting to visit or to have a friend or relative be. . .a patient. (P30, Board member)

These emotional and cultural pressures motivated clinical staff to seek an immediate way to control hyperactive symptoms, sometimes through medication,

So, you look at the patient, everybody’s so anxious around, the staff, including the relative, because it’s really traumatic if it is a severe hyperactive delirium, and are trying to climb out of bed, they’re pulling their catheters and, yeah, fighting and whatever, and, yes, they expect you to do something to remove that as quickly as possible. (P11, Doctor)

I think they really struggle to see somebody restless and, and agitated and immediately want to settle that down, usually through giving medication. (P03, Doctor)

In contrast, patients with hypoactive delirium were likely not to be recognised by clinical staff,

I think that we’re bad at picking up the hypoactive ones; so, when people are actually quite quiet we probably don’t pick up those things, we might be asking them how they’re feeling but, as I say, I think they’re the ones that tend to get missed. (P15, Doctor)

Patients with hypoactive symptoms did not disrupt the calm environment or provoke emotional urgency to act,

The hypoactive patients, there may be people who are sitting very quietly and placidly and not ‘causing any trouble’ and therefore they don’t necessarily get the same degree of attention as somebody who is, you know, being very loud and confused and wandering. (P20, Doctor)

There’s less pressure. . .she’s quite quiet, isn’t she, so she’s not causing a problem; and that’s where I think it’s not always acted on. (P25, Doctor)

In terms of COM-B, participants’ emotional responses, part of ‘automatic motivation’, powerfully influenced their focus on managing hyperactive symptoms, including medication use. This was compounded by a cultural necessity to maintain a calm environment, an influence related to ‘social opportunity’.

**2.** **Varied understanding of delirium influences the reactive focus on managing hyperactive symptoms (Psychological capability/reflective motivation)**

When staff and volunteers responded to patients’ distressing behaviours, many were not acting in the context of an understanding of delirium as a medical condition with a structured process of care. Understanding of delirium recognition, prevention and management, varied between individuals and staff groups. This strongly influenced both the reactive focus on managing hyperactive symptoms, and the management itself. Most health care assistants, nurses, therapists, non-clinical staff and volunteers reported little or no previous training, whereas most doctors reported learning opportunities about delirium.

a. Under-recognition of delirium as a medical condition

Nurses, HCAs, therapists, volunteers and some doctors mostly described hyperactive symptoms and behaviours, or used other terms such as ‘confusion’ or ‘terminal agitation’. They rarely described hypoactive symptoms.

I think probably a lot of the time people use other words. . .they don’t tend to use the word delirium that much. (P15, Doctor)

It depends. . . what the main thing is that they’re presenting with, if its hallucinations then it’ll just be they’re hallucinating or they’ve been a little muddled. . .agitated. (P1, Physiotherapist)

Some doctors emphasised the importance of naming delirium to promote understanding of it as a medical condition requiring a structured response,

I refer to it as delirium, but in the past, I have referred to it as confusion, acute confusional state, different things, but now I feel that it’s really important to label it as such. . . so that people understand. . . there’s often a significant reversible element to it and by naming it. . . we can then look to investigate it, reverse what may be reversible, and try and help that person better. So I think it’s important to. . .give it a, a label as. . . a medical condition, because. . .it elevates its importance and emphasises it and allows us to take a certain type of action. (P20, Doctor)

b. ‘I don’t think you can. . .prevent it really’: Lack of shared understanding of delirium prevention.

Most HCAs and volunteers and some nurses and therapists did not express knowledge of the potential for delirium prevention.

I don’t think you can, can you, prevent it really. (P04, HCA)

Some nurses and therapy staff expressed limited knowledge of ways of preventing delirium, but these were not systematically implemented. Some doctors clearly articulated a range of strategies but it is likely that HCAs and nurses would primarily be responsible for many of these tasks.

Having clear signage on the doors, helping patients to be able to find their way around so they’re more orientated, getting dressed during the day, pyjamas at night. . . other preventative things. Keeping people well hydrated, well nourished, keeping on top of constipation, on top of urinary tract infections by keeping people well hydrated; those kinds of things. (P18, Doctor)

It was highlighted that some of these tasks may be done during usual care, without awareness of their relevance for delirium prevention,

Specific prevention, I don’t think we’re particularly aware of. I think obviously in the hospice it’s easier to make sure patients are getting plenty of drinks, we review patients’ medications most days; so, all those side of things I think we’re good at. So, I think probably without being aware of it we’re preventing it but we’re just not saying we’re preventing it. (P03, Doctor).

The lack of a shared team understanding of delirium as a medical condition, with hypoactive as well as hyperactive symptoms, which may be prevented, contributed to staff’s reactive focus on hyperactive symptom management.

The influence of participants’ understanding on *how* they managed delirium was also explored.

c. Varied understanding of modifiable causes of delirium

The extent of clinicians’ understanding of causes of delirium, and their modification potential, varied. This influenced their management; most HCAs and volunteers, and some nurses and therapy staff expressed understanding of limited possible causes of ‘confusion’, including medication and infections. Some nurses and therapy staff described a range of modifiable causes, and the doctors articulated more detailed knowledge of predisposing and precipitating factors,

There’s a number of factors why our patients are more at risk of having delirium. They obviously have. . .a significant illness that is life limiting, and on top of that. . .they might be having numerous . . .medical treatments. . .it might be that a patient has developed an infection. . . it might be that there’s some biochemical abnormalities, so you might do some bloods. . . looking at renal failure, liver failure, that might just push somebody over that threshold into. . . a delirious state. . . often drugs. . . that might be interacting and causing a delirium. . .that might be reversible if we removed those. (P20, Doctor)

When patients were nearing death, some clinicians considered hyperactive symptoms to be ‘terminal agitation’, or assumed that only symptom control was now appropriate,

Terminal agitation. . .so, medication is the only really; lots of reassurance, lots of support. (P22, Nurse)

She did look right poorly, we did think she was near the end of life, so we didn’t look for any underlying causes. (P15, Doctor)

Some doctors described combining their knowledge of modifiable causes with judgement of the likely benefit or burden of investigations in decision-making,

If it’s something reversible, is it appropriate that we treat that?. . .because. . . if we’re in the last days then it might not be. . . Are they in the last days of life? Right, OK different scenario, let’s not take those bloods. . . they might have high calcium but we’re not going to treat that because actually they’re not going to get the benefit. (P25, Doctor)

One doctor highlighted the value of continuing less intrusive investigations close to the end of life,

Unless. . . we knew we were in the last hours of life, I think we would always be looking for reversible causes because constipation can happen at any point and is quite a common cause. . . we’d still be wanting to try and use non-pharmacological approaches, reverse, look for anything that we could do. (P18, Doctor)

In terms of COM-B, by engaging in active clinical reasoning regarding the benefits and burdens of addressing modifiable causes of delirium, they combined psychological capability with reflective motivation in their decision-making.

d. Understanding of the role of medication for delirium symptom management

Many of the doctors expressed knowledge of recent evidence which does not support routine medication use for delirium. They, and some nurses, described taking a cautious approach to using medication, considering non-pharmacological approaches first,

We don’t always use first line medication. . . We like to look at other things first. Is it the environment as well?. . . Rather than just going with medication (P14, Nurse)

However, many nurses described using antipsychotics and benzodiazepines routinely to ‘settle’ hyperactive patients,

Well initially just help him relax, maybe some lorazepam, if that would help; longer-term diazepam. . .look at the analgesia, something to help him settle at night; and it might be that he needs some antipsychotics. (P19, Nurse).

The doctors’ knowledge of the evidence interacted with their beliefs that medication could sometimes be effective in reducing distressing symptoms (reflective motivation). They described difficulty in balancing the benefits and risks,

I think it’s managing the extreme agitation and distress that does need medication. . . it’s very easy, potentially, to get into a spiral of sedation which then makes them very sleepy during the day which then makes the night-time worse. So I think we try to be very conscious of that, but equally it’s very hard to not be doing that at night if they’re at risk or very, very distressed and agitated. (P03, Doctor)

e. The need for a shared team understanding of delirium care

We found variation between staff groups in their understanding of delirium recognition, prevention and management which strongly influenced their reactive focus on symptom management. The need to develop a shared team understanding was identified to enable a structured approach to delirium care,

If you say. . .if this is happening call it delirium and if its delirium these are the steps you take then a common approach. (P11, Doctor)

This domestic worker’s comment highlights that training should include all staff and volunteers with direct patient contact,

If we had training on how to approach people like that and maybe say morning to you. . .but then come out with a load of gobblygooch. . .it can be a bit hard. . .how do you approach that person? (P12, Domestic worker)

A shared team understanding would support a structured approach to delirium care, including prevention and early recognition, as well as management.

**3.** **Hospice culture and environment influence person-centred delirium care (Social and Physical opportunity)**

Person-centred and family-centred approaches to delirium care were both supported and challenged by hospice cultural norms (social opportunity) and staffing levels (physical opportunity).

a. ‘Patients and their families are at the centre’: Person-centred and family-centred approach to delirium care (Social opportunity)

Participants involved in direct care reported making efforts to engage with, and calm, patients who were confused or agitated through learning about their lives and interests and person-centred activities,

Their history, their work history, their hobbies, family. . . if they’ve got a pet. The more you have on that, the more normal things we can talk to a patient about that they. . . may be able to remember and relate to. (P19, Nurse)

When I went into his room I saw a classic car magazine. . .I said, ‘I’m involved with that and we restore Austin Healeys’ and his eyes lit up and he said, ‘Buick Healey?’ I said, ‘That’s right. . .I’ll bring you some books in’. I do try and find. . .common ground with people. (P24, Volunteer)

Participants providing direct care described a range of reassuring interpersonal strategies including a calm tone of voice, non-threatening body language and sensitive use of touch. They encouraged families to bring in familiar items to help orientate and reassure patients,

I’d mentioned. . . to bring in some photographs and put them on a memory board and make him familiar that this is his family. . . and then I suggested that his family maybe do a tape of their voices so that on a night. . . it was a ritual where he would get in his bed and. . . he was in and out, in and out, he couldn’t settle, and then once we put the headphones on and. . . his tape and the voices of his family, he seemed to settle and relax. (P26, HCA)

Many participants identified that the presence of a family member could help calm patients, although they recognised this could be stressful for the family,

We. . .appreciate that (*family*) carers, they use this as their respite as well when the patients are in, but particularly if people know they’re unsettled overnight sometimes they do stay if they know that makes them more feel at home and more comfortable and less agitated. (P1, Physiotherapist)

Staff described how providing families with information and support could reduce their distress and help them to support the patient,

We find that by people knowing what’s going on, their anxieties drop, so it. . . has an effect on the patient. . . so it’s. . . like a calming circle. . . it’s just such a lot better for the patient. (P14, Nurse)

Person-centred and family-centred care were highly valued, not only by clinical staff but throughout the organisation,

The patients and their families are at the centre of everything that the hospice does. (P30, Board member)

However, participants identified challenges in providing person-centred care with people with hyperactive delirium, including staff stress (theme 1) and the intensive staffing levels required (theme 3). When patient behaviours conflicted with needs of other patients and their families, the needs of others were sometimes prioritised.

We would maybe move that patient if he was maybe upsetting other people. (P14, Nurse)

We would probably go with Haloperidol if we felt that he was getting to the point where he might harm himself or. . . cause distress to other patients. (P18, Doctor)

If they are more wakeful at night and you’re trying to use other (*person-centred, non-pharmacological*) strategies. . .have you got the staffing levels there to be able to support that? Particularly in an environment like this where it could be disruptive to somebody else that’s maybe acutely at end of life and you’re trying to manage them, and their relatives, it can be quite distressing to have somebody wandering into rooms. (P1, Physiotherapist)

b. ‘If we do need extra staff we usually do get extra staff’: Time and staffing levels enable person-centred delirium care (Physical opportunity)

Participants felt hospice staffing levels generally enabled them to spend time with delirious patients, using person-centred calming strategies, and contrasted this with hospital settings,

The ratio of nurses to patients here is, we have less patients to each nurse, so I do think you can give that better quality, and I think in the hospitals they’re so pushed. (P8, Nurse)

Bringing extra staff on. . .that’s the. . .difference between that and hospital. . .Hospital, you can have. . .security men sitting at the end of the bed and they looked after a lorry park before. . .and they obviously, they don’t get that connection with them, do they?. . . sometimes they antagonise them more really, because they don’t have that understanding, do they?. . .I found that quite, quite distressing really. (P16, HCA)

Nonetheless, when there were many highly dependent patients, and at night-time, this became more difficult,

I think it works well when there’s maybe just one or two people like that and the staff can dedicate time, but when, if all. . .beds are filled and there are a lot of other really unwell people, people who are maybe imminently dying, then it is harder to spend that amount of time that’s maybe needed with someone who has a delirium. (P15, Doctor)

Night-time tends to be a time when things seem to be really difficult, and whether that’s because it is night-time, there’s less staff, as well as symptoms. . .being more difficult. (P03, Doctor)

Time and staffing pressures were an influence on use of medication to manage hyperactive symptoms,

The easiest answer is to go to the drug cupboard. . .I think if there’s more pressures on peoples’ time. . . it’s that quick fix, isn’t it? (P15, Doctor)

The need for higher, flexible staffing levels when working with patients with delirium was recognised at management level, although cost was identified as a potential barrier,

We’re very lucky here because if we do need extra staff we usually do get extra staff. . .we actually speak to the senior sisters and we’ll look at our dependencies, we’ll look at what each individual needs. . .we have a lot of flexibility. (P14, Nurse)

There is a higher intensity of staff required. So from a management point of view. . .the fact that the patients have become more complex, nursing establishments based on years ago just don’t fit anymore. So. . .it definitely costs more, and I think that’s something that hospices, that’s going to be a challenge as time goes on. (P09, Manager)

Some volunteers contributed to care of delirious patients by reassuring and spending time with them, although they highlighted that their role must have clear limits,

I’m just hopefully. . . somebody. . . to reassure and. . .somebody to talk to. . . and perhaps in a way, when I’m here, I have more time to do that than. . .the qualified staff. (P23, Volunteer)

In terms of COM-B, the physical opportunities of adequate time, staffing levels and volunteer input, supported at organisational level, were important enablers for person-centred delirium care.

c. Supportive multidisciplinary team working (social opportunity)

Clinical staff and volunteers described a culture of multidisciplinary team working which supported management of hyperactive delirium through peer support, team problem-solving and reflective learning (social opportunity). For example, nurses and HCAs took turns with patients with hyperactive delirium to reduce staff stress. All team members’ contributions to clinical problem-solving were valued,

Just because you’re a senior member of staff, it doesn’t mean that you know, it might be one of the care assistants that. . .has said, oh well I’ve noticed this. . .So it’s about. . .working together as a team. (P05, nurse)

I think we’re a good supportive team so we bounce off each other so we’re more, well, have we tried this, could we do this. . . and we’ll look at things and see if we’ve covered the bigger picture before we start to intervene. (P32, HCA)

Structured opportunities for support, reflective practice and learning were provided and valued at clinical and management level,

Trying to provide an environment. . .where people are supported. . .giving people time to reflect on those particularly difficult situations. . .so we’ve got reflective practice, clinical supervision and then team days; and I firmly believe that. . . these sort of challenges, we can only build up a sort of resilience and a team that can cope, and not just cope but can manage that, if they have that opportunity to. . .share their concerns and fears. (P09, Manager)

## Discussion

### Main findings of study

Our study found that staff and volunteers focused on managing hyperactive symptoms. Delirium prevention, early recognition and hypoactive delirium, received much less attention. A powerful motivating influence for this reactive approach, including the use of medication, was participants’ emotional responses (automatic motivation) in managing the impact of hyperactive symptoms on patients and their families.

Knowledge and understanding of delirium care, along with training opportunities (psychological capability), varied between staff groups. Under-recognition of delirium as a medical condition, and poor knowledge of the potential for delirium prevention, strongly influenced this reactive focus. When patients had delirium close to death, some clinicians assumed only symptom control was appropriate, while others maintained active clinical reasoning (reflective motivation) regarding the benefits and burdens of investigating modifiable causes, for example, treatment of constipation or medication review.

Staff and volunteers’ management of hyperactive symptoms was supported by the cultural norm of valuing person and family-centred approaches. However, hyperactive symptoms which disrupted the norm of a calm hospice environment, challenged person-centred care (social opportunity). Adequate time and staffing levels (physical opportunity) and supportive team working (social opportunity) supported this approach.

### Strengths and limitations

Strengths of this study are our diverse participants; robust data collection and analysis. Our use of COM-B enabled us to carry out structured analysis of the influences on delirium practice which can inform the design of behaviour change interventions.^35^ Despite including only two UK hospices, transferability is increased as our findings can be compared with those of other delirium studies using the COM-B framework.^43^ A limitation of using qualitative interviews was that participants may not have identified preventative behaviours carried out during usual care, as they were unaware of their relevance to delirium.

### What this study adds

Our findings suggest that to improve delirium care in inpatient hospices, a shift ‘upstream’ in practice is needed, with increased focus on delirium prevention and early recognition. There has been only preliminary research into delirium prevention in palliative care settings.^8,9^ A pilot study, reporting low adherence to a delirium prevention intervention, found that clinicians were more strongly motivated to enact responsive care than anticipatory.^44^ Our theoretically informed analysis of the influences on this ‘reactive approach’ can be used to inform further research to develop effective interventions.

Our findings regarding the influence of psychological capability, align with and build upon previous interview studies with palliative care nurses which found limited understanding of delirium recognition, assessment and management.^20–22^ Hosie et al.^21^ found variation in nurses’ use of delirium terminology, capability to frame symptoms as delirium and conduct comprehensive assessment. Importantly, we also identified a need for increased understanding of the potential for delirium prevention, particularly for staff most likely to carry out the relevant strategies.

Due to our range of participants, our study highlighted the variation in knowledge between different staff and volunteer roles, with doctors having more learning opportunities and understanding than others involved in direct patient care. A previous systematic review recommended inter-professional education for complex delirium care.^44^ There is a need to develop a shared team understanding to enable a structured approach to delirium care; reflected by recent calls for effective and strong interdisciplinary collaboration in this field.^45^

Our finding that staff’s emotional responses (automatic motivation) are important in driving their focus on managing hyperactive symptoms, including the use of medication, is consistent with the results of an Australian survey study, which found that 82% of palliative care clinicians reported emotional influences, including patient, family and staff distress, on their delirium treatment behaviours.^43^ Delirium educational approaches need to address the emotional impact of delirium, as well as clinical learning or more technical needs.^46^ Behaviour change theory can inform intervention design to both increase delirium knowledge and engage staff’s emotional responses to motivate practice change.

Behaviour change techniques could be used to increase staff and volunteers’ understanding of the potential health and emotional consequences of increased practice focus on delirium prevention and early recognition: reducing delirium incidence, severity and distress. Other emotionally engaging behaviour change techniques could include: the use of ‘credible sources’ to deliver delirium education (e.g. senior staff; external experts; people with lived experience), and ‘modelling’, such as reflective case study-based learning.^38^

As guidelines primarily recommend non-pharmacological management,^16,17^ our findings regarding supportive social and physical opportunities are important to inform clinical practice and service provision. Enablers included a cultural norm of valuing person-centred and family-centred care throughout the organisation. However, this was challenged when clinical decision-making, including medication use, was influenced by the disruption caused to others by hyperactive symptoms. In the UK context, the high value placed upon maintaining the cultural norm and perception of hospices as calm and welcoming, may also partly be influenced by their reliance on charitable fundraising. Our previous qualitative literature synthesis found that, although some patients and families may wish for sedation to be used to achieve comfort and a ‘peaceful’ death, others prefer to be able to communicate, despite the delirium.^2^ Further, sedation may mask hyperactive symptoms and ‘convert’ patients to hypoactive delirium,^47^ experiencing as much distress as those with hyperactivity.^4,7^ Interventions combining education on medication use with reflective learning on complex treatment decision-making, including whose needs are primarily being addressed, would build upon the person-centred approach to delirium care.

The delirium prevention pilot study in palliative care units, found that a culture of compassionate care and interdisciplinary collaboration supported the intervention.^44^ We found that supportive team working and organisational support were important and may need further strengthening. Lack of time or manpower inhibits the use of non-pharmacological strategies in favour of medication use.^25^ We found favourable staffing levels compared to hospital settings but an increase was needed with highly dependent patients, especially at night-time. The cost implications of this may be challenging in the context of the cost-of-living crisis and COVID-related financial pressures on hospices. Harnessing the skills of suitably trained and supported volunteers may become increasingly important.

In conclusion, our theoretically informed study identified influences on delirium practice behaviours in hospices, including strengths and areas for change. Further research, using ethnographic observation methods, would be valuable to increase understanding of practice relevant to delirium prevention during usual care. Our study findings can be used to inform clinical practice and research to develop behaviour change interventions, tailored to hospice settings, which aim to improve delirium care and reduce the distress that it causes.

## Supplemental Material

sj-pdf-1-pmj-10.1177_02692163231170655 – Supplemental material for ‘It’s tough. It is hard’: A qualitative interview study of staff and volunteers caring for hospice in-patients with delirium Click here for additional data file. Supplemental material, sj-pdf-1-pmj-10.1177_02692163231170655 for ‘It’s tough. It is hard’: A qualitative interview study of staff and volunteers caring for hospice in-patients with delirium by Imogen Featherstone, Najma Siddiqi, Lesley Jones, Eleonora Coppo, Trevor Sheldon, Annmarie Hosie, Anna Wolkowski, Shirley H Bush, Johanna Taylor, Andrew Teodorczuk and Miriam J Johnson in Palliative Medicine
