# Supplementary material for: ‘It’s tough. It is hard’: A qualitative interview study of staff and volunteers caring for hospice in-patients with delirium
Source: Palliat Med. 2023 May 2;37(7):993–1005. doi: 10.1177/02692163231170655 (PMC10320705; doi:10.1177/02692163231170655)
Supplement: sj-pdf-1-pmj-10.1177_02692163231170655 – Supplemental material for ‘It’s tough. It is hard’: A qualitative interview study of staff and volunteers caring for hospice in-patients with delirium [file sj-pdf-1-pmj-10.1177_02692163231170655.pdf]

Supplementary file 1:

Interview Schedule: Healthcare Professional

- Introduction to researcher and project
- Check has read and understood the information sheet and allow time to discuss any questions or concerns (Sent with introductory letter/email)
- Check participant happy for audio recording and use of anonymised quotes
- Consent form to sign (according to GCP)

Introductory question:

1. Have you come across delirium in your work at the hospice? Could you tell me more about that? (*Understanding of delirium; terms used e.g. terminal restlessness, terminal agitation, acute confusion*)

Case Studies:

- Give participant case study 1 to read and ask them the case study questions. Then repeat with case study 2.

2. Is there anything about this that reminds you of a situation you have come across at work in the hospice? (*If no, ask questions hypothetically*)
3. Can you tell me about this situation? (describe how patient was behaving?)
4. What did you do?

   (*Recognition, assessment, management of delirium- investigating causes, symptom control*)

   What did others do?
5. How did you feel about the situation?
   a. Were there aspects of the situation that you found challenging? / Unnerving?
6. How did you see your role in this situation? (*team working, communication*)
7. In looking back at that situation, is there anything you would do differently?

After case study 2:

8. Are there any patients you've worked with who have behaved in ways that remind you of both case studies? *(mixed delirium)*

Further questions:

9. Is there anything that you do that can help to prevent delirium from developing?
10. What would help to support you in caring for patients with delirium?
11. Have you had any training or education on delirium? Are there particular aspects of delirium care that you would like to learn more about?
12. What would you say to someone starting out, when they see someone with delirium for the first time?

13. Have you ever used any tools or documentation related to delirium care?

What do you think of these ideas about how to treat delirium?

- Delirium symptoms often treated using antipsychotic medication or sedation.
- But some recent research suggests that antipsychotics may not be effective (not reducing how long symptoms last or how severe they are)
- Sedation can stop patients from being able to communicate.
- Some research suggests it could be better to target the causes of delirium to treat it and also to prevent it. By, for example, screening patients for delirium; making sure they are drinking plenty of fluids; reminding them where they are; reviewing their medication; investigating and treating infections and other causes.

14. What do you think about this?
15. Are there parts of this approach that you already do routinely?
    a. What benefits might there be, if this type of approach was implemented in the hospice?
    b. What challenges might there be?
16. What would need to change to put this into practice regularly?
17. Is there anything that we haven't covered so far, that you would like to talk about before we finish?

Case study 1: Hyperactive delirium

Edward is an 89 year old librarian with prostate cancer. He has been admitted from home for pain control. His opioid analgesia has been titrated rapidly over the past few days and his pain has been responding well. However, the night staff reported very disturbed behaviour last night. He appeared very frightened and agitated, shouting loudly for help, but also using very abusive language and hitting out at the nurses. The patients in the adjacent rooms were wakened by him and were frightened he would come into their rooms. The next door patient's wife complained saying her husband should be able to spend his last few days in peace, not be in fear of his life. His family are horrified, saying he would never use such language, or be aggressive to anyone.

Case study 2: Hypoactive delirium

Mrs Wright, aged 86, has mesothelioma. She was admitted to hospital for drainage of a pleural effusion and transferred two days ago to your hospice for rehabilitation and management of breathlessness. Her family report that apart from the few days before her hospital admission, when her breathlessness had become worse, she was independent at home with the support of meals on wheels. Following her drainage, she developed a chest infection and was given antibiotics. She was also prescribed Lorazepam which hospital staff said settled her and helped her to sleep in the noisy ward.

Since admission to the hospice she has been quiet and stayed in her room. She does not seem to know where she is, and the physiotherapist has reported that she has not engaged with breathlessness management techniques. At handover, the night staff report that they have heard her mumbling to herself in the evenings, but it is hard to make out what she is saying.
